
JOURNAL INFORMATION
==============================
NLM Title Abbreviation: Wirtschaftsdienst
Journal ID: Wirtschaftsdienst
NLM Journal ID: 101086612

Wirtschaftsdienst (Hamburg, Germany : 1949)

ISSN: 0043-6275

ARTICLE INFORMATION
==============================
PMCID: PMC8052196
PMID: 33897054
DOI: 10.1007/s10273-021-2882-9
Article ID: 2882
Article version: 1
Subjects: Kurz Kommentiert

Impfabsichten: Sie müssen umgesetzt werden!

Vaccination intentions: They must be turned into action!

Stauf Julia julia.stauf@behavia.de

Schubert Manuel manuel.schubert@behavia.de

Reitemeyer Anja anja.reitemeyer@behavia.de

Behavia - Behavioral Public Policy and Economics GmbH, Onckenstr. 18, 12059 Berlin, Deutschland
Electronic publication date: 2021 Apr 17
Print publication date: 2021
Volume: 101
Issue: 4
First page: 245
Last page: 245
Copyright: © Der/die Autor:in(nen) 2021
License: Open Access: Dieser Artikel wird unter der Creative Commons Namensnennung 4.0 International Lizenz veröffentlicht (https://creativecommons.org/licenses/by/4.0/deed.de).
Open Access wird durch die ZBW — Leibniz-Informationszentrum Wirtschaft gefördert.
License URL: https://creativecommons.org/licenses/by/4.0/

issue-copyright-statement: © The Author(s) 2021

==============================

In der Diskussion über die Gründe für den schleppenden Fortschritt der Impfkampagne wird neben der Wirksamkeit und Sicherheit der Impfstoffe und den Problemen mit Verfügbarkeit, Logistik und Organisation auch das Verhalten der Bevölkerung thematisiert. Dabei werden vor allem Faktoren betrachtet, welche die Impfbereitschaft von Personen beeinflussen können. Ein zentrales Problem wird dabei aber bislang übersehen: Menschen setzen ihre Pläne häufig nicht in die Tat um.

Hinter der Entscheidung zur Impfung steht eine individuelle Abwägung von Nutzen und Risiken.[1] Wie Risiken wahrgenommen werden, hängt dabei nicht nur von der reinen Faktenlage ab, sondern wird stark durch das soziale Umfeld und die mediale Berichterstattung beeinflusst. Zudem muss ein potenzieller Impfling genügend Vertrauen in die Hersteller, die Zulassungsprozesse und das medizinische Fachpersonal haben. Nur dann wird er sich dazu entschließen, sich impfen zu lassen.

Die Impfbereitschaft allein reicht aber noch nicht aus. Der Entschluss muss in die Tat umgesetzt werden. Sobald es die Priorisierung erlaubt, muss im Dickicht von Online-Formularen und Hotlines ein Termin ergattert werden. Danach folgt die Planung – Vorgesetzte müssen benachrichtigt, Kinderbetreuung muss sichergestellt oder der Anfahrtsweg organisiert werden. Und selbst die beste Planung scheitert oft an der Ausführung. Wie oft werden Vorsorgeuntersuchungen verschoben, weil etwas anderes dazwischenkommt oder sie schlicht vergessen werden?

Wenn Menschen Schwierigkeiten haben, ihren Absichten Taten folgen zu lassen, spricht man in der Verhaltenswissenschaft von einem Intention Action Gap. Diese Schere zwischen Vorsatz und Ausführung gibt es in allen Lebensbereichen, und sie ist so menschlich wie verbreitet.[2] Hier droht ein Problem, das bislang wenig Aufmerksamkeit erhalten hat: Es ist nicht ungewöhnlich, wenn von denen, die angeben, sich impfen lassen zu wollen, am Ende nur die Hälfte geimpft ist (z. B. Auslander et al., 2019). Vor dem Hintergrund der anhaltenden Schwierigkeiten, überhaupt genügend Impfstoff zur Verfügung zu stellen, mögen die individuellen Hindernisse bei der Umsetzung der Impfabsichten trivial erscheinen. Trotzdem sollte ihre Wirkung auf den künftigen Impffortschritt nicht unterschätzt werden.

Die WHO hat bereits Ende Dezember 2020 eine Reihe von Vorschlägen gemacht, wie die Nachfrage nach Impfungen gefördert werden kann. Neben vertrauensbildenden Maßnahmen und Möglichkeiten, konstruktiv mit Bedenken umzugehen, geht es bei diesen Vorschlägen vor allem darum, die technischen und logistischen Hürden auf dem Weg zur Impfung abzubauen (WHO, 2020). Die individuellen Gründe für den Intention Action Gap mögen vielfältig sein, aber es gibt eine sehr einfache und effektive Gegenmaßnahme: Je leichter es Menschen gemacht wird, ihre Absichten umzusetzen, desto höher ist die Erfolgswahrscheinlichkeit.

Hier stehen Bund und Länder in der Pflicht. Sie müssen nicht nur die notwendigen Voraussetzungen schaffen, damit Menschen sich impfen lassen können, sondern sollten darüber hinaus dafür sorgen, dass dies so einfach und komfortabel wie möglich ist. Die Nutzerfreundlichkeit muss oberste Priorität haben, die Verfahren müssen auf die Bedürfnisse der verschiedenen Zielgruppen zugeschnitten sein. Jeder Impfwillige sollte mit ein paar Klicks oder einem Anruf einen Termin in der Nähe buchen können – ohne Warteschleifen, dafür aber mit automatischen Erinnerungen. Sonst haben wir irgendwann gut ausgestattete Impfzentren mit ausreichend Impfstoff, aber keiner geht hin.

1 Für eine ausführliche Darstellung von Kosten und Nutzen siehe z. B. Wein (2021).

2 Für eine Diskussion der Gründe und möglichen Gegenstrategien siehe Sheeran und Webb (2016).


Literatur

Auslander B A Meers J M Short M B Zimet G D Rosenthal S L A qualitative analysis of the vaccine intention-behaviour relationship: parents’ descriptions of their intentions, decision-making behaviour and planning processes towards HPV vaccination Psychology & Health 2019 34 3 271 288 10.1080/08870446.2018.1523408 30406692 PMC6476672
Sheeran P Webb T L The intention-behavior gap Social and personality psychology compass 2016 10 9 503 518 10.1111/spc3.12265
Wein T Ist eine Impfpflicht gegen das Coronavirus nötig? Wirtschaftsdienst 2021 101 114 120 10.1007/s10273-021-2852-2 33642648 PMC7896173
World Health Organization (WHO) (2020), Behavioural considerations for acceptance and uptake of COVID-19 vaccines: WHO Technical Advisory Group on Behavioural Insights and Sciences for Health, meeting report, 15. Oktober 2020, https://www.who.int/publications/i/item/9789240016927 (28. Februar 2021).
